# LncRNA miR-17-92a-1 cluster host gene (MIR17HG) promotes neuronal damage and microglial activation by targeting the microRNA-153-3p/alpha-synuclein axis in Parkinson’s disease

**DOI:** 10.1080/21655979.2022.2033409

**Published:** 2022-02-09

**Authors:** Jianzhong Zhang, Yun Yang, Chaoyang Zhou, Ronglan Zhu, Xiang Xiao, Bin Zhou, Dengfeng Wan

**Affiliations:** a Department of Neurosurgery, Jiangxi Provincial People’s Hospital Affiliated to Nanchang University, Nanchang, Jiangxi, China; b Department of Neurology, Jiangxi Provincial People’s Hospital Affiliated to Nanchang University, Nanchang, Jiangxi, China

**Keywords:** Parkinson’s disease, MIR17HG, miR-153-3p, alpha-synuclein, inflammation

## Abstract

Long noncoding RNAs (lncRNAs) have been regarded as modulators of neurodegenerative diseases. Here, we addressed the role of lncRNA miR-17-92a-1 cluster host gene (MIR17HG) in Parkinson’s disease (PD). C57BL/6 mice and SH-SY5Y cells were intervened with 6-hydroxydopamine (6-OHDA) to set up PD models *in vivo* and *in vitro*. Quantitative reverse transcription-polymerase chain reaction (qRT-PCR) was implemented to compare the expression of MIR17HG and miR-153-3p. Cell viability and apoptosis were estimated by 3-(4,5-dimethyithiazol-2-yl)-2,5-diphenyl-tetrazolium bromide (MTT) and Western blot (WB). The expression of alpha-synuclein (α-syn, SNCA) in BV2 was validated by enzyme-linked immunosorbent assay (ELISA). Reactive oxygen species (ROS) generation and lactate dehydrogenase (LDH) and superoxide dismutase (SOD) activity were evaluated using commercially available kits. Bioinformatics analysis, the dual-luciferase reporter assay, RNA immunoprecipitation (RIP) and qRT-PCR were conducted to demonstrate the interactions between miR-153-3p, MIR17HG, and alpha-synuclein (SNCA). MIR17HG was up-regulated while miR-153-3p was down-regulated in PD patients, mouse models and cells. Inhibiting MIR17HG attenuated neuronal apoptosis, microglial activation and SNCA expression in PD mice. Conditioned medium from 6-OHDA-treated SH-SY5Y cells intensified microglial inflammation, while inhibition of MIR17HG or overexpression of miR-153-3p restrained the inflammatory responses. MIR17HG’s function was enforced by sponging miR-153-3p and releasing the attenuation of the putative targets of miR-153-3p and SNCA. Overall, MIR17HG, by targeting miR-153-3p and up-regulating SNCA, stimulates neuronal apoptosis and microglial inflammation in PD.

## Graphical Abstract

**Figure:**
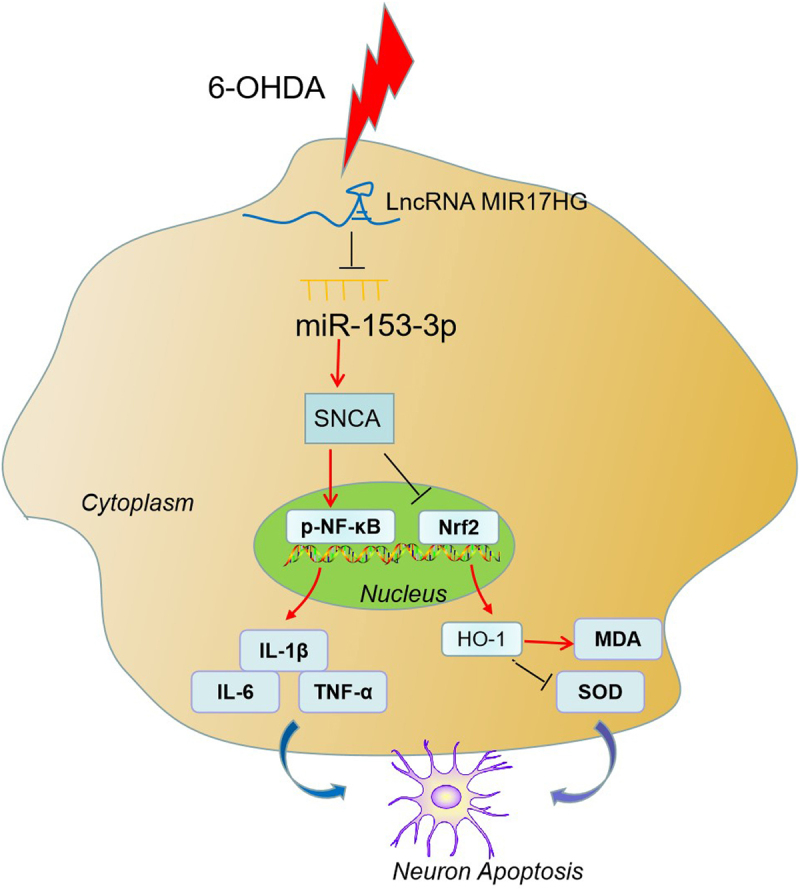

## 1. Introduction

Parkinson’s disease (PD) is the main contributor to neurodegenerative disorders in the elderly [1]. The clinical manifestations of PD are motor and non-motor deficits [2], and its motor symptoms are usually related to the death of dopamine neurons in the substantia nigra pars compacta (SNpc) and the production of Lewy bodies containing alpha-synuclein (α-syn, SNCA) [2]. On the other hand, microglia-mediated oxidative stress and neuroinflammation trigger degeneration of dopaminergic neurons [3]. Emerging evidence suggests that abating microglial inflammation may be an effective way to treat inflammatory diseases [4]. In parallel, molecular mechanisms play a crucial regulatory role in inflammation, while their role in PD is unclear.

Long noncoding RNAs (lncRNAs) contain over 200 nucleotides and cannot be translated into proteins. Multiple reports have manifested that lncRNAs are implicated in regulating immune response, carcinogenesis, stress reaction, cell development and differentiation at the transcriptional, epigenetic or post-transcriptional levels [5]. Also, several studies have stated that lncRNAs contribute to neurodegenerative diseases [6–9]. The LncRNA miR-17-92a-1 cluster host gene (MIR17HG) is a long noncoding RNA located at 13q31.3 and is 927 bp in length. According to reports, MIR17HG can act as a tumor-suppressive gene or oncogene to influence the progression of non-small cell lung cancer [10] and colorectal cancer [11]. Nonetheless, it is still unknown about the function of MIR17HG in PD.

MicroRNAs (miRNAs) are small noncoding RNAs that modulate gene expression post-transcriptionally by restraining messenger RNA (mRNA) translation or facilitating mRNA degradation [12]. A slew of miRNAs is dysregulated in PD, including miR-30, miR-29, let-7, miR-485 and miR-26 [13]. As a miRNA, miR-153-3p is down-regulated in oxygen–glucose deprivation/reoxygenation (OGD/R)-induced neurons in neurological disorders, and miR-153-3p overexpression eases OGD/R-mediated neuronal injury [14]. Of special note, the miR-153-3p level is lowered in an *in-vitro* PD model induced by 1-methyl-4-phenylpyridinium (MPP+) in SH-SY5Y cells, and miR-153-3p down-regulation declines SH-SY5Y cells’ viability and amplifies their apoptosis [15]. Accordingly, we inquired into the function and specific mechanism of miR-153-3p in PD.

alpha-synuclein (SNCA), located on chromosome 4q21-23, is an α-syn associated with familial PD and highly expressed in the SN, thalamus, hippocampus, amygdala and caudate nucleus of the brain. The accumulation of α-syn is a pathological hallmark of PD and is linked to a reduction in neuronal synapse length and axonal degeneration in midbrain dopaminergic neurons [16]. As reported, plasma contents of Interleukin-1β (IL-1β) and total α-syn are higher in PD patients than in controls, and both IL-1β and α-syn contents are positively linked to the severity of motor injury in PD patients [17]. Thus, SNCA is highly expressed in PD and correlates with the severity of PD. On the other hand, classical theories hold that lncRNAs act as competitive endogenous RNAs (ceRNAs) to sponge miRNAs, which in turn modulate α-syn expression [18]. Up-regulation of lncRNA-p21 sponges miR-1277-5p and indirectly elevates the α-syn level, thereby curbing MPP-treated SH-SY5Y cells’ viability and accelerating apoptosis [19]. Nonetheless, it is not clear about the pathogenic mechanism of LncRNA MIR17HG and miR-153-3p/SNCA in PD.

Here, we inquired into the impacts of lncRNA MIR17HG on the viability, apoptosis, inflammatory factors and oxidative stressors in 6-hydroxydopamine (6-OHDA)-treated mice and 6-OHDA-induced SH-SY5Y cells. Moreover, we uncovered further mechanisms of SH-SY5Y cell survival and apoptosis. MIR17HG and SNCA were found to be up-regulated and miR-153-3p was down-regulated in PD patients, mouse models and cells. Meanwhile, there is a targeting regulatory association between MIR17HG, miR-153-3p and SNCA. It is therefore hypothesized that LncRNA MIR17HG may stimulate neuronal apoptosis and microglial inflammation in PD by targeting miR-153-3p to up-regulate SNCA. Our research may shed light on a new direction for the treatment of PD.

## 2. Methods and materials

### 2.1. Clinical sample selection and manipulations

Blood samples were pooled from 40 PD patients (onset within 24 hours) who were admitted to Jiangxi Provincial People’s Hospital Affiliated to Nanchang University between April 2018 and April 2019 and from 20 healthy controls. The cells were removed out of the blood samples by low-speed centrifugation, and the plasma was gathered and frozen in a − 80°C refrigerator. This research was granted by the Medical Ethics Committee of Jiangxi Provincial People’s Hospital Affiliated to Nanchang University. All sample providers agreed to participate in this study and subscribed to the consent form.

### 2.2. Animals

All tests were authorized by the Ethics Committee of Jiangxi Provincial People’s Hospital Affiliated to Nanchang University (Approval number: JXPHE-2020-021-19) and complied with the National Institute of Health guidelines for the care and use of animals. Twenty C57BL/6 mice (half male and half female, 22–24 g of body weight, 8 weeks old) were ordered from the Animal Center of the Chinese Academy of Sciences (Shanghai, China). Mice were confined in temperature- and humidity-controlled areas with a 12-hour light/dark cycle. The experiments were done in strict accordance with national regulations and the EC Council Directive (86/609/EEC) and the Principles for the Care of Laboratory Animals (NIH Publication No. 85–23) on the protection of animals utilized for scientific and experimental purposes.

### 2.3. The mouse PD model

Mice were deeply anesthetized through intraperitoneal administration of sodium pentobarbital (40 mg/kg) and were immobilized in a stereotactic frame. Craniotomy was implemented using a dental drill, and 4 μL of 6-hydroxydopamine (6-OHDA; H4381, 3 μg/μL, Sigma Aldrich, St. Louis, MO, USA) dissolved in sterile saline containing 0.02% ascorbic acid was stereotactically injected into the right substantia nigra (SN) (from bregma: AP, −3.0 mm; ML, −1.2 mm; DV, −4.7 mm) at a flow rate of 1 μL/minute. In the control group, sterile saline containing 0.02% ascorbic acid was injected into the right SN [20]. After the drug administration, the syringe was left in place for 10 minutes and then slowly pulled out. With the skin sutured, the mouse was withdrawal from the stereotaxic, maintained on a heating pad for 30 minutes, and then backtracked to the cage. In the mouse model, 6-OHDA was progressively lost to dopaminergic neurons in the SN as it was taken up by neuronal terminals in the striatum and translocated to dopaminergic neurons, resulting in injury to dopaminergic neurons. On the other hand, we injected lentiviruses of si-NC and si-MIR17HG (total 2 μL, 2.1 × 10^7^ TU/mL) purchased from Shanghai Gene Pharma Co., Ltd (Shanghai, China) into the right SN at 0.5 μL/minute after 6-OHDA treatment [21].

### 2.4. Apomorphine (APO)-induced rotation test

Drug-induced rotational behavior is usually adopted to determine the extent of unilateral lesions [22]. Each mouse was administered with APO (0.25 mg/kg in 0.9% saline, intraperitoneally) and put in a stainless-steel cylindrical bowl. The net number of rotations (contralateral turns minus ipsilateral turns) was counted over a 30-minute period 5 minutes after the APO intervention. After injection of 6-OHDA, an APO-induced rotation test was performed to assess the therapeutic effect at weeks 2, 4, 6 and 8, respectively. The assessment was implemented by observers who were not aware of animal preconditioning.

### 2.5. Rotarod test

The rotarod test [23], which was designed to assess alterations in motor coordination following the administration of drugs that caused sedation or muscle relaxation, was conducted using a device consisting of a wooden pole with a non-slip surface. Mice were trained by placing them on a rotating bar (7 rpm/minutes) prior to the 6-OHDA injection. Testing was performed at week 2 post-operatively. Mice were placed on a rotating bar (7 rpm/minutes) and the number of falls/minutes was recorded.

### 2.6. Passive avoidance test

The mouse’s learning and memory performance was evaluated with the passive avoidance test [24]. The instrument rooms utilized in this experiment included a compartment with low illuminance and a white illumination room. During the acquisition/conditioning phase, all mice were placed in the white compartment. When the mouse crossed to the black compartment, it would receive a mild electric shock to the foot (0.5 mA, 1 s). From this, the mouse learned that moving to the dark compartment had negative consequences, and it passively avoided entering a dark compartment. The initial latency (IL) of mice entering the dark room was recorded, excluding mice with IL S&gt;60s. At week 6 post-injury, each mouse was put in an illuminated chamber for assessment of passive avoidance responses. The time interval between placing the mice in the illuminated chamber and their entry into the dark chamber was monitored using step latencies (STL) (maximum cutoff time of 180 s).

All behavioral observations were carried out between 9 am and 12 pm. After the last behavioral test, the mice were euthanized with carbon dioxide and their brains were gathered for biochemical analysis.

### 2.7. Sample collection

Mice received euthanasia with sodium pentobarbital (150 mg/kg) at week 8 after 6-OHDA injection. The mouse heart was rapidly perfused with approximately 200 mL of saline followed by approximately 300 mL of 4% paraformaldehyde (PFA). The craniotomy was applied for brain tissue extraction and isolation. Fresh nigrostriatal tissues were quickly removed from the rat brain using ophthalmic forceps. The SN of the brains of PD and control mice was harvested and secured in 4% paraformaldehyde for 24 hours and then frozen in 30% sucrose. The brain was coronally sectioned to a thickness of 30 μM after embedding and stored at −20°C for relevant testing [25].

### 2.8. Tissue immunofluorescence

Frozen sections of fresh tissues were secured in 4% formaldehyde solution for 10 minutes, permeabilized with Triton X.100 for 15 minutes, and closed with immunostaining solution for 1 hour. They were then successively maintained with the primary antibody IBA1 (1:100, ab178847, Abcam, MA, USA) overnight at 4°C in the refrigerator and with the secondary antibody for 1 hour at room temperature (RT) and dyed with 4ʹ6-diamidino-2-phenylindole (DAPI) for 3 to 5 minutes. Fluorescence microscopic observation was performed following sealing [26].

### 2.9. Immunohistochemistry (IHC)

The number of tyrosine hydroxylase (TH)- and SNCA-positive cells was counted by IHC [27]. Paraffin-embedded nigrostriatal tissue sections were subjected to dewaxing in xylene and hydration with gradient ethanol. Paraffin sections were soaked in 10 mm citrate buffer (pH 6.0) and boiled in a pressure cooker at 121°C for 4 minutes for antigen recovery. The slides were then allowed to cool at RT, immersed in a 10 mm citric acid buffer and flushed with suitable amounts of PBS. The endogenous peroxidase activity was choked by treating the sections with 3% H_2_O_2_ for 15 minutes at RT. Sections were maintained overnight at 4°C with the primary antibody SNCA (1:150, ab138501, Abcam, MA, USA) and the primary antibody TH (1:250, ab156008, Abcam, MA, USA), followed by the incubation with the secondary antibody for 0.5 hours at RT. Immunohistochemical staining was made with 3,5-diaminobenzidine (DAB, Fuzhou, Maxim), with the sections viewed under a light microscope and photographed. Immunohistochemical staining was quantified with the Image-Pro-Plus 6.0 software (Media Controlnetics, Inc., Rockville, MD, USA).

### 2.10. Cell culture

Neuroblastoma cell line SH-SY5Y and microglia BV2 were ordered from the American Type Culture Collection (ATCC, Rockville, MD, USA). Cells were grown in the DMEM-F12 medium comprising 10 mmol/L Hepes, 10% fetal bovine serum (FBS, HyClone, Logan, UT, USA), and a 1% mixture of penicillin and streptomycin, and kept at 37°C with 5% CO_2_ and saturated humidity. The medium was substituted every 2–3 days, and the cells were sub-cultured every 4–5 days. SH-SY5Y cells were intervened with 200 μM of 6-OHDA (dissolved in ascorbic acid; Sigma, St. Louis, MO, USA) for 24 hours to set up an *in-vitro* PD model [28]. The conditioned medium (CM) was obtained from 6-OHDA-induced SH-SY5Y cells and then co-cultured with BV2 cells for 24 hours to assay the impact of 6-OHDA on BV2 cells [29].

### 2.11. Cell transfection

SH-SY5Y and BV2 at the logarithmic growth stage were taken, trypsinized, sub-cultured and grown in 6-well plates (5 × 10^6^/well). After cell growth had stabilized, the siRNA negative control for MIR17HG (si-NC), siRNA for MIR17HG (si-MIR17HG), vectors, MIR17HG overexpression plasmids, miR-153-3p mimics and miR-153-3p inhibitors and the paired negative counterparts were transfected into the above cells. Cells were kept at 37°C with 5% CO_2_. After 24 hours of transfection, total cellular RNA was isolated for quantitative reverse transcription-polymerase chain reaction (qRT-PCR) to validate the alterations in miR-153-3p levels in the cells.

### 2.12. Quantitative reverse transcription-polymerase chain reaction (qRT-PCR)

Total RNA extraction from tissues and cells was routinely performed by utilizing the TRIzol (Invitrogen, Carlsbad, CA, USA) method [30]. After the purity test, miRNA, LncRNA, and mRNA underwent reverse transcription into cDNA using the mirPremier® microRNA Isolation Kit (Sigma, St. Louis, MO, USA) and the PrimeScript RT kit (Invitrogen, Shanghai, China), respectively. Then, we adopted the SYBR®Premix-Ex-Taq™ (Takara, TX, USA) and the ABI7300 system for qRT-PCR. GAPDH served as a housekeeping gene to verify the mRNA levels of LncRNA MIR17HG, SNCA, IL-1β, interleukin-6 (IL-6), TH, and tumor necrosis factor (TNF-α). In contrast, U6 served as that of miR-153-3p. The primer sequences for each molecule are as follows. MIR17HG: F: 5’-TGTGCAGATTGAGCTCTCCT-3’, R: 5’-TCCTGACAAAATGCAGCCTG-3’; miR-153-3p: F: 5’-AATCGGCGTTGCATAGTCACAAA-3’; R: 5’-CAGTGCAGGGTCCGAGGT-3’; SNCA: F: 5’-GACTGGGCACATTGGAACTG-3’; R:5’-TGCCTGTGGATCCTGACAAT-3’; GAPDH: F: 5’-CTCCTCCTGTTCGACAGTCAGC-3’; R: 5’-CCCAATACGACCAAATCCGTT-3’. IL-1β: F: 5’-TCATCTTTTGGGGTCCGTCA-3’; R: 5’-GGCTCATCTGGGATCCTCTC-3’; IL-6: F: 5’-TTTCACCAGGACCGTCTCTCCT-3’; R:5’-AGACAGCCACTCACCTCTTC-3’; TNF-α: F: 5’-ATCCCAGGTTTCGAAGTGGT-3’; R: 5’-TCTGGGCAGGTCTACTTTGG-3’; TH: F: 5’-GCGTGGACAGCTTCTCAATT-3’; R: 5’-TGTTCCAGTGCACCCAGTAT-3’. U6: F: 5’-CTCGCTTCGGCAGCACATATACTA-3’; R:5’-ACGAATTTGCGTGTCATCCTTGC-3’.

### 2.13. Western blot (WB)

WB was adopted to gauge protein expression in PD mice’s nigrostriatal tissues and 6-OHDA-induced BV2 cells and neuronal cells [31]. SH-SY5Y, BV2 and transfected cells were dissociated with RIPA lysis solution (Beyotime Biotcchnology, Shanghai, China) and then subjected to protein extraction. Next, a 5% spacer gel and a 10% separation gel were taken and 20 μL of protein samples were sampled. After electrophoresis, the proteins were transferred to PVDF membranes (Millipore, Bedford, MA, USA) for 90 minutes at a constant current of 200 mA. Subsequently, 5% skimmed milk powder was adopted to seal the membranes, which were maintained overnight at 4°C with primary antibodies. Afterward, the membranes were flushed with TBST the following morning and maintained with horseradish peroxidase (HRP)-tagged anti-rabbit secondary antibody (Abcam, ab6721, concentration 1:1000, MA, USA) for 1 hour at RT, rewashed and exposed with ECL (Millipore, Bedford, MA, USA). The primary antibodies used here including anti-TH antibody (Abcam, ab156008, 1:1000, MA, USA), anti-Bax antibody (Abcam, ab32503, 1:1000), anti-Bcl2 antibody (Abcam, ab185002, 1:1000), anti-C-Caspase3 antibody (Abcam, ab13847, 1:1000), anti-SNCA antibody (Abcam, ab0138501, 1:1000), anti-iNOS antibody (Abcam, ab15191, 1:1000), anti-COX2 antibody (Abcam, ab179800, 1:1000), anti-NF-κB antibody (Abcam, ab32536, 1:1000), anti-p-NF-κB antibody (Abcam, ab76302, 1: 1000), anti-Nrf2 antibody (Abcam, ab62352, 1: 1000), anti-HO-1 antibody (Abcam, ab13243, 1: 1000), and anti-GAPDH antibody (Abcam, ab9485, 1:1000).

### 2.14. Enzyme-linked immunosorbent assay (ELISA)

BV2 cells were inoculated at 2 × 10^5^ cells/well in 24-well plates. After 24 hours, the supernatant of BV2 cells was harvested and centrifuged at 1000 rpm for 10 minutes at 4°C. The supernatant was taken after centrifugation. Brain tissues (100 mg) from PD mice were added to 0. 01 mol of phosphate buffer saline (PBS) solution at 0. 05 g/mL, homogenized manually to make 10% tissue homogenate and then centrifuged at 2000 rpm for 8 minutes at low temperature. After that, the precipitate was discarded and the supernatant was reserved. The expression of the inflammatory factors interleukin-1β (IL-lβ), interleukin-6 (IL-6) and tumor necrosis factor-α (TNF-α) in BV2 microglia and PD mice’s brain tissues was evaluated using ELISA kits (Multisciences (Lianke) Biotech, Co. Ltd) [32].

### 2.15. Detection of oxidative stress

Superoxide dismutase (SOD) activity and malondialdehyde (MDA) levels were gauged with commercially available test kits (Jiancheng, Nanjing, China). SN tissues and cells were homogenized in ice-cold PBS and centrifuged to obtain supernatants for SOD and MDA assays as instructed by the manufacturer. MDA contents are expressed as mmol/L protein, while SOD activity is expressed as a percentage [33].

## 2.16. 3-(4,5-dimethyithiazol-2-yl)-2,5-diphenyl-tetrazolium bromide (MTT) assay

Cell viability was monitored using the MTT assay (Boster Bioengineering Co., Ltd., Wuhan, China) [34]. SH-SY5Y cells were diluted to 4 × 10^4^ cell/mL suspensions with the complete medium and added to 96-well culture plates (200 μL/well). Following 24 hours, a large proportion of the cells were adherent to the wall, and the primary medium was substituted with 0.5% FBS for cell synchronization. Twenty-four hours later, the culture medium was aspirated. With the completion of 24, 48 and 72 hours of incubation, 10 μL of 5 mg/mL MTT solution was added to each well and kept for 4 hours at 37°C in an incubator with 5% CO_2_ and saturated humidity. Next, the medium was discarded, and each well was filled with 150 μL of dimethyl sulfoxide (DMSO) and maintained for 10 minutes with shaking. After the dissolution of the precipitate, the optical density (OD) values were reviewed at 570 nm with a microplate reader.

### 2.17. Flow cytometry (FCM)

SH-SY5Y cells’ apoptosis was gauged with the Annexin V-fluorescein isothiocyanate (FITC)/propidine iodide (PI) staining kit (BD Biosciences, San Diego, CA, USA) [15]. In a nutshell, Cells were flushed twice with PBS, added to 400 μL of pre-cooled PBS and then incubated with 10 μL AnnexinV-FITC and 5 μL PI respectively for 30 minutes at 4°C protected from light. After that, apoptosis was immediately assayed by FCM, and the apoptotic cell percentage was computed after processing by computer software.

### 2.18. BrdU staining

Cell proliferation assessment was made by the BrdU Cell Proliferation Assay Kit [35]. SH-SY5Y cells were seeded in 96-well plates at 6 × 10^4^ cells/mL and kept overnight at 37°C with 5% CO_2_. 20 μL BrdU (WuHan AmyJet Scientific Inc. Wuhan, China) was added to each well and further incubated for 12 hours. Then, the stationary liquid was added and incubated at RT for 30 min. After being flushed with PBS, the cells were incubated with pre-diluted detected antibodies at RT for 1 hour and then maintained with HRP-tagged secondary antibody for 1 hour. Subsequently, the stop buffer was added, and the positive cells were reviewed and calculated by utilizing a fluorescence microscope.

### 2.19. Lactate dehydrogenase (LDH) release assay

The cells or tissue samples were homogenized, and the supernatant was removed by centrifugation at low speed. The LDH delivered to the cells was monitored using the kit as per the manufacturer’s guidelines (Beyotime Biotechnology, Shanghai, China). The OD was gauged at 490 nm. The amount of lactate released after cell lysis in relation to the total lactate release was determined [36].

### 2.20. LUC experiment

All luciferase reporter vectors (MIR17HG-WT, MIR17HG-MUT, SNCA-WT, and SNCA-MUT) were constituted by Promega Corporation (Madison, WI, USA). SH-SY5Y and BV2 cells (4.5 × 10^4^) were seeded in 48-well plates and cultured to 70% confluence. MIR17HG-WT, MIR17HG-MUT, SNCA-WT, or SNCA-MUT was then cotransfected with miR-153-3p mimics or negative controls in these cells by employing Liposome 2000. Following 48 hours, the luciferase activation was tested following the manufacturer’s directions. All tests were made in triplicate and repeated three times.

### 2.21. RNA immunoprecipitation (RIP)

The EZ-Magna RIP RNA Binding Protein Immunoprecipitation Kit (Millipore, Billerica, MA, USA) was employed to estimate the binding specificity between miR-153-3p and MIR17HG, miR-153-3p and SNCA [15]. Briefly, SH-SY5Y cells or BV2 cells were rinsed in PBS and lysed in RIP lysis buffer comprising protease and ribonuclease inhibitors. Then, 100 μL of cell lysates were maintained with the RIP buffer comprising magnetic beads coupled to the human anti-Argonaut 2 antibody (Ago2; Millipore) or standard mouse immunoglobulin G (IgG; Millipore). The mixture was then co-cultured with Proteinase K to break down the proteins and facilitate the isolation of immunoprecipitated RNA. qRT-PCR was undertaken to check the levels of MIR17HG and SNCA in the precipitates.

### 2.22. Data analysis

The SPSS 24.0 statistical software was applied to process the data. Measures were manifested as mean ± standard deviation (x ± s). Statistical analysis was made by adopting the GraphPad Prism 7 software. Student *t*-test was used for comparing two-group data. For multiple group comparisons, one-way ANOVA was used with Bonferroni posttest for comparisons between selected two groups. * *P* < 0.05, ** *P* < 0.01, *** *P* < 0.001.

## 3. Results

In this study, we first detected the expression features of MIR17HG, miR-153-3p and SNCA in PD patients and healthy donors, as well as in PD mouse model. Then we performed both in vitro and in vivo experiments for confirming the functions of MIR17HG on neuron apoptosis, microglial activation, and PD progression. The targeted relationship between MIR17HG and miR-153-3p, miR-153-3p and SNCA were verified. Then rescue experiments were performed for confirming the regulatory effects of MIR17HG-miR-153-3p-SNCA axis in neuron and microglia.

### 3.1. Expression characteristics of LncRNA MIR17HG, SNCA and miR-153-3p in PD patients

**Figure 1.**
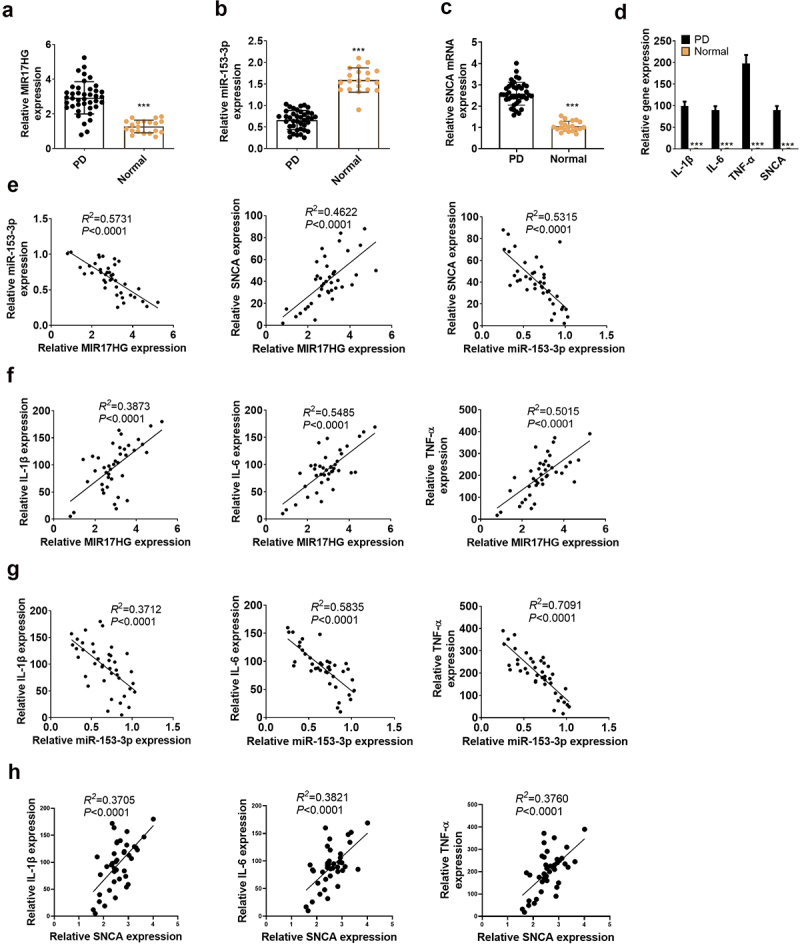
Expression features of LncRNA MIR17HG and miR-153-3p in PD patients.

To figure out the expression features of MIR17HG, miR-153-3p and SNCA in PD patients and healthy populations, we implemented qRT-PCR. As a result, by contrast with the normal population (N = 20), lncRNA MIR17HG and SNCA were up-regulated, while miR-153-3p was down-regulated (*P* < 0.001, Figure 1a-c) in the plasma of PD patients (N = 40). Also, the expression characteristics of IL-1β, IL-6, TNF-α and SNCA in PD patients and normal populations were gauged by qRT-PCR. The outcomes uncovered that the profiles of the above molecules were uplifted in the plasma of PD patients (*P* < 0.001, Figure 1d). As evidenced by Pearson analysis, lncRNA MIR17HG was adversely related to miR-153-3p (*R*^2^ = 0.5731, *P* < 0.0001), lncRNA MIR17HG was positively correlated with SNCA (R^2^ = 0.4622, *P* < 0.0001), and miR-153-3p was negatively associated with SNCA (*R*^2^ = 0.5315, *P* < 0.001, Figure 1e). Besides, MIR17HG was positively linked to IL-1β (*R^2^ *= 0.3873, *P* < 0.0001), IL-6β (*R^2^ *= 0.5485, *P* < 0.0001), and TNF-a (*R^2^ *= 0.5015, *P* < 0.0001, Figure 1f). In contrast, miR-153-3p was reversely correlated to IL-1β (*R^2^ *= 0.3712, *P* < 0.0001), IL-6 (*R^2^ *= 0.5835, *P* < 0.0001), and TNF-a (*R^2^ *= 0.7091, *P* < 0.0001) (Figure 1g). In parallel, SNCA was positively related to IL-1β (*R^2^ *= 0.3705, *P* < 0.0001), IL-6 (*R^2^ *= 0.3821, *P* < 0.0001), and TNF-a (*R^2^ *= 0.3760, *P* < 0.0001) (Figure 1h). Thus, high expression of MIR17HG or low expression of miR-153-3p contributed to PD.

(a-c): qRT-PCR analyzed the profiles of LncRNA MIR17HG, miR-153-3p and SNCA in PD (N = 40) and in the normal population (N = 20). *** *P* < 0.001 (vs.Normal group). (d): Detection of IL-1β, IL-6, TNF-a expression by qRT-PCR in PD patients and normal controls. *** *P* < 0.001 (vs.Normal group). €: Pearson correlation analysis was conducted to determine the relationship between MIR17HG and miR-153-3p, MIR17HG and SNCA, and miR-153-3p and SNCA. *R*^2^ = 0.5731, *P* < 0.0001. *R*^2^ = 0.4622, *P* < 0.0001. *R*^2^ = 0.5315, *P* < 0.0001. (f): Pearson was employed to analyze the relationship between MIR17HG and IL-1β, IL-6, TNF-a and SNCA. *R*^2^ = 0.3873, *P* < 0.0001. *R*^2^ = 0.5485, *P* < 0.0001. *R*^2^ = 0.5015, *P* < 0.0001. (g): Pearson was employed to analyze the relationship between miR-153-3p and IL-1β, IL-6, TNF-a. *R*^2^ = 0.3712, *P* < 0.0001. *R*^2^ = 0.5835, *P* < 0.0001. *R*^2^ = 0.7091, *P* < 0.0001. (h): Pearson analysis of the relationship between SNCA and IL-1β, IL-6, and TNF-a in the plasma of PD patients. *R^2^ *= 0.3705, *P* < 0.0001. *R^2^ *= 0.3821, *P* < 0.0001. *R^2^ *= 0.3760, *P* < 0.0001.

### 3.2. Expression features of LncRNA MIR17HG, SNCA and miR-153-3p in the PD model

**Figure 2.**
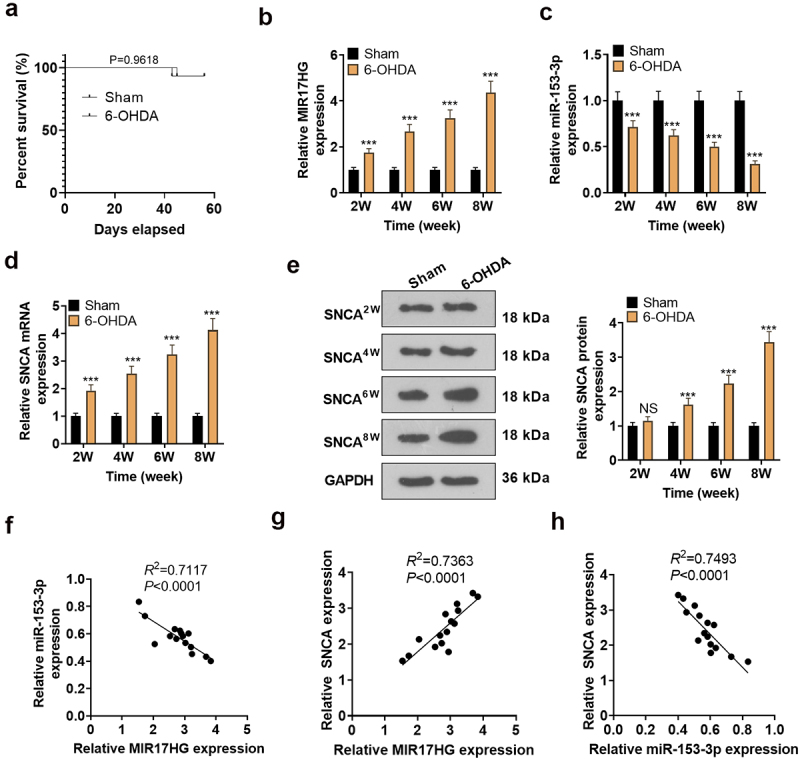
Expression features of LncRNA MIR17HG, SNCA and miR-153-3p in the PD model.

We constructed a PD mouse model through ventricular injection of 6-OHDA. The overall survival rate of PD group and sham group showed no significant difference. We checked the expression of lncRNA MIR17HG, miR-153-3p and SNCA in the SN of PD mice using qRT-PCR and WB at weeks 2, 4, 6 and 8. It was concluded that compared to the Sham group, 6-OHDA-induced PD mice had up-regulated MIR17HG (*P* < 0.001, Figure 2b) and down-regulated miR-153-3p (*P* < 0.001, Figure 2c), accompanied by enhanced mRNA and protein expression of SNCA (*P* < 0.001, Figure 2d, e). At week 8, using Pearson analysis, we discovered a reverse correlation between MIR17HG and miR-153-3p (*R*^2^ = 0.7117, *P* < 0.0001, Figure 2f) and a positive correlation between MIR17HG and SNCA (*R*^2^ = 0.7363, P < 0.0001, Figure 2g). Meanwhile, miR-153-3p was reversely related to SNCA (*R^2^ *= 0.7493, *P* < 0.0001, Figure 2h).

(a). The overall survival rate of mice in PD group and sham group was counted. (b-d): Expression of LncRNA MIR17HG, miR-153-3p and SNCA in the SN region of PD mice was checked using qRT-PCR at weeks 2, 4, 6 and 8 after ventricular injection of 6-OHDA. (e): Protein profiles of SNCA in the SN region of PD mice were evaluated using WB at weeks 2, 4, 6 and 8 after ventricular injection of 6-OHDA. *** *P* < 0.001 (vs.Sham group). (f-h): The relationships between MIR17HG and miR-153-3p (F), MIR17HG and SNCA (G), and miR-153-3p and SNCA (H) were analyzed using Pearson at week 8 after ventricular injection of 6-OHDA. *R^2^ *= 0.7117, *P* < 0.0001. *R^2^ *= 0.7363, *P* < 0.0001. *R^2^ *= 0.7493, *P* < 0.0001.

### 3.3. *Attenuating MIR17HG weakened neuronal apoptosis in the PD model* in vitro *and* in vivo

**Figure 3.**
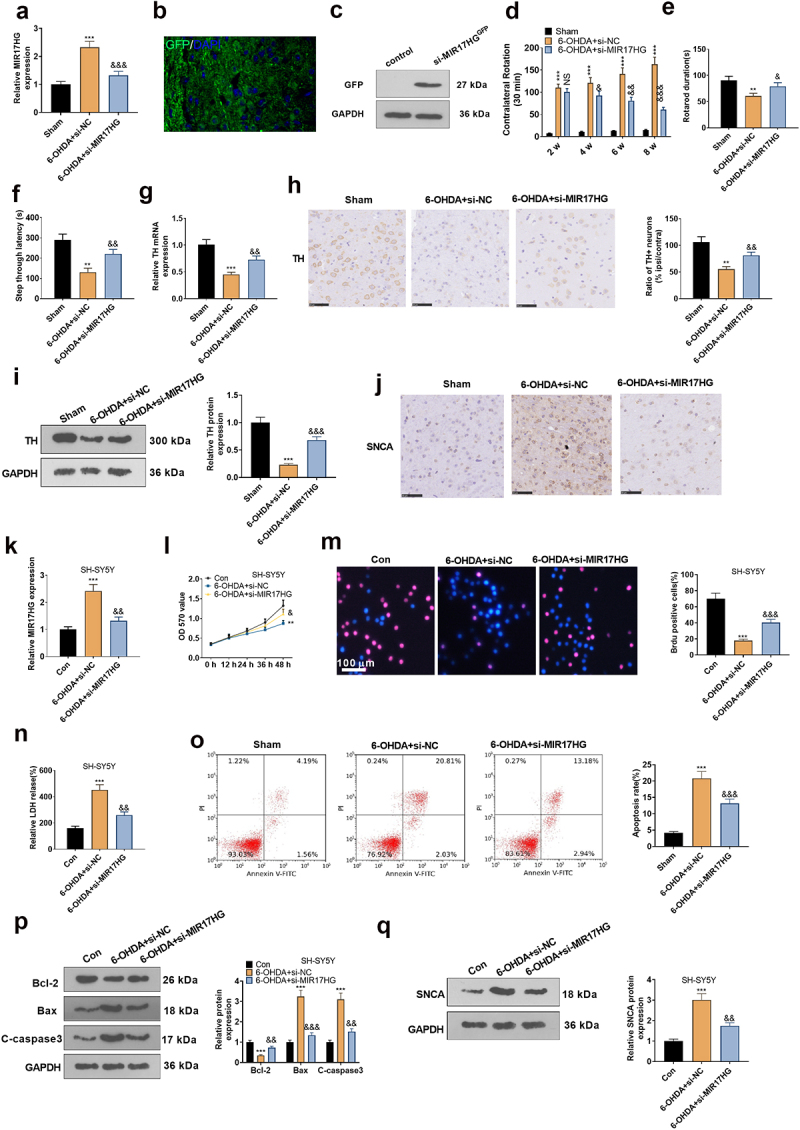
Attenuating MIR17HG mitigated neuronal apoptosis in the PD model *in vitro* and *in vivo.*

To make certain the impact of MIR17HG on PD, we set up a PD mouse model using 6-OHDA and injected the model mice with si-MIR17HG lentivirus. qRT-PCR outcomes demonstrated that si-MIR17HG restrained the MIR17HG expression in the brain tissue of PD mice versus the 6-OHDA+si-NC group (*P* < 0.05, Figure 3a). The lentiviral particles carrying GFP were injected into the brain. Fluorescence assay and Western blot showed successful lentiviral infection of si-MIR17HG (Figure 3b-c). Besides, the behavioral performance of PD mice was assessed using the APO-induced rotation test, Rotarod test and passive avoidance test. It was discovered that in comparison to the Sham group, PD mice displayed an increase in the number of rotations and falls within 30 minutes and a shortening of the interval from the illuminated chamber to the dark chamber after 6-OHDA injection. The addition of Si-MIR17HG lentivirus substantially reduced the number of rotations and falls on the turnbuckle within 30 minutes and prolonged the interval time between mice’ going from the illuminated chamber to the dark chamber versus the 6-OHDA group (*P* < 0.05, Figure 3d-f). As a marker of DA neurons, TH exhibits pronounced expression in dopaminergic neurons, which synthesizes the catecholamines necessary for dopamine generation [37]. Hence, we examined TH mRNA and TH cell ratios in the SN with qRT-PCR and IHC. The data demonstrated that 6-OHDA induced a down-regulation of the mRNA and cellular ratio of TH, which was reversed by si-MIR17HG (*P* < 0.05, Figure 3g, h). Consistent with the IHC results, WB affirmed that the TH protein expression was choked in the 6-OHDA group versus the sham group, while it was boosted after inhibition of MIR17HG (*P* < 0.05, Figure 3i). As testified by IHC, the SNCA profile was amplified in the 6-OHDA group versus the sham group, whereas it was notably decreased by MIR17HG inhibition (*P* < 0.05, Figure 3j). These outcomes substantiated that 6-OHDA caused motor dysfunction and intensified neuronal apoptosis in mice, and MIR17HG hampered this effect of 6-OHDA. *In vitro*, a MIR17HG knockdown model was founded by transfecting si-MIR17HG into SH-SY5Y cells (*P* < 0.05, Figure 3k), followed by treatment with 6-OHDA for 24 hours. Then MTT, BrdU staining, LDH kit, FCM and WB were employed to check neuronal proliferation, LDH release, and the expression of apoptosis-related proteins and SNCA. As expected, by contrast with the 6-OHDA+si-NC group, abating MIR17HG notably strengthened neuronal viability (*P* < 0.05, Figure 3l,m), reduced LDH release (*P* < 0.05, Figure 3n), intensified cell apoptosis (Figure 3o), lowered the expression of Bax and Caspase3, elevated the level of Bcl2 (*P* < 0.05, Figure 3p), and up-regulated SNCA (*P* < 0.05, Figure 3q). Taken together, attenuating MIR17HG eased neuronal apoptosis in PD both *in vivo* and *in vitro*.

Lentiviral Si-MIR17HG was injected into 6-OHDA-treated mice, and SH-SY5Y cells were transfected with Si-MIR17HG to suppress the MIR17HG expression. (a): qRT-PCR assessed MIR17HG expression in brain tissues of PD mice. (b): FISH assay was applied to monitor the location and level of GFP after GFP-marked si-MIR17HG probe injection. (c). Western blot was performed for detecting GFP. (d-f): The effects of MIR17HG inhibition on the behavioral profile of PD mice were examined using APO-induced rotational behaviors (d), the rotarod bar test (e), and the passive avoidance assay (f). G and H: qRT-PCR and IHC tested the expression of TH mRNA and TH-positive cell mass. (i): WB exhibited alterations in TH protein expression in brain tissues of PD mice. (j): The SNCA expression was gauged by IHC. **P < 0.01, ***P < 0.001 (vs.Sham group), &*P* < 0.05, &&*P* < 0.01, &&&*P* < 0.001 (vs.6-OHDA+Si-NC group). (k): qRT-PCR analyzed the relative expression of MIR17HG in SH-SY5Y cells. (l): The viability of transfected SH-SY5Y cells was monitored by MTT. (m): BrdU staining counted the number of BrdU-positive cells in transfected SH-SY5Y cells. (n): The LDH kit was applied to determine LDH levels in SH-SY5Y cells. (o): Cell apoptosis was tested by FCM. (p and q): WB detection of apoptosis-related protein expression (Bax, Bcl2, and Caspase3) and SNCA expression in SH-SY5Y cells. **P < 0.01, *** *P* < 0.001 (vs.Con group), && *P* < 0.01, &&& *P* < 0.001 (vs.6-OHDA+Si-NC group).

### 3.4. *Attenuation of MIR17HG mitigated microglial inflammation in PD models* in vivo *and* in vitro

**Figure 4.**
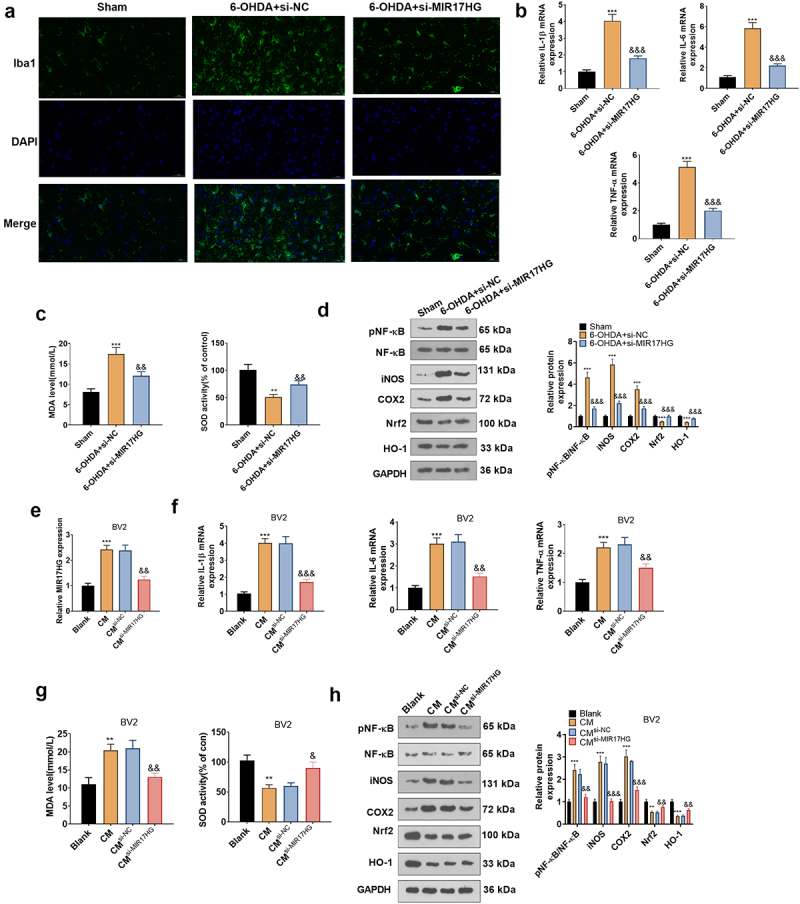
Inhibition of MIR17HG curbed the inflammatory response in microglia in PD models both *in vivo* and *in vitro.*

Microglial activation is implicated in dopaminergic neuronal loss in a 6-OHDA-induced mouse PD model. The IBA1-positive cell number mirrors the percentage of motivated microglia as IBA1 is a marker of microglial activation. The IBA1 profile in the PD mouse model was assessed by tissue immunofluorescence, which disclosed that 6-OHDA elevated the IBA1-positive cell number in the SN versus the Sham group, whereas knockdown of MIR17HG reduced IBA1-positive cells (*P* < 0.05,Figure 4a). In parallel, qRT-PCRexhibited that knockdown of MIR17HG distinctly choked the expression of IL-1β, IL-6 and TNF-α enhanced by 6-OHDA (*P* < 0.05, Figure 4b). What’s more, MDA and SOD levels in the SN of PD mice were measured using MDA and SOD kits. The outcomes uncovered that in comparison to the Sham group, 6-OHDA substantially elevated MDA contents and choked the SOD activity, whereas knockdown of MIR17HG declined MDA levels and facilitated SOD activity (*P* < 0.05, Figure 4c). The pro-inflammatory cytokines TNF-α, IL-1β and IL-6 and the pro-inflammatory enzymes iNOS and COX-2 contribute to the inflammatory process. In parallel, the nuclear factor carotenoid 2-related factor 2/heme oxygenase 1 (Nrf2/HO-1) signal is a multi-organ protective chain that prevents oxidative stress damage [38]. Thus, we probed the impact of MIR17HG on the NF-κB/iNOS/COX2 and Nrf2/HO-1 pathways. As demonstrated by WB outcomes, 6-OHDA activated the phosphorylation of NF-κB (p65), boosted the expression of COX2 and iNOS and curbed the levels of Nrf2 and HO-1 versus the Sham group. In contrast, knocking down MIR17HG hindered the phosphorylation of NF-κB (p65) and the levels of COX2 and iNOS and boosted the expression of Nrf2 and HO-1 (*P* < 0.05, Figure 4d). In summary, hindering MIR17HG attenuated microglial activation and protected dopaminergic neurons in the mouse PD model induced by 6-OHDA. Again, we examined the MIR17HG inhibition’s influence on BV-2 cells to figure out its anti-inflammatory mechanism in cells. The CM was collected from 6-OHDA-induced SH-SY5Y cells and co-cultured for 24 hours with BV2 cells transfected with si-NC and si-MIR17HG. qRT-PCR uncovered that the MIR17HG profile was amplified in the CM^6-OHDA^ group versus the Blank group. BV2 cells transfected with si-MIR17HG had decreased MIR17HG expression versus the CM^6-OHDA+si-NC^ group (*P* < 0.05, Figure 4e). The mRNA expression of IL-1β, IL-6, and TNF-α and the levels of MDA and SOD in BV2 cells were measured by qRT-PCR and MDA and SOD kits. It turned out that the contents of IL-1β, IL-6 and TNF-α and the content of MDA increased and the SOD activity declined in the CM^6-OHDA^ group versus the Blank group. Inhibition of MIR17HG markedly choked IL-1β, IL-6, and TNF-α expression and MDA levels and increased SOD activity in BV2 cells versus the CM^6-OHDA+si-NC^ group (*P* < 0.05, Figure 4f,g). It is well-established that the NF-κB/iNOS/COX2 and Nrf2/HO-1 pathways contribute to inflammation and oxidative stress. Therefore, we tested the impact of MIR17HG inhibition on NF-κB (P65) phosphorylation and pro-inflammatory enzyme protein (COX2 and iNOS) expression in BV-2 cells using WB. The outcomes revealed that the expression of Nrf2 and HO-1 was impeded and the levels of COX2 and iNOS were facilitated in the CM^6-OHDA^ group versus the Blank group, accompanied by enhanced phosphorylation of NF-κB (p65). Compared to the CM^6-OHDA+si-NC^ group, MIR17HG knockdown restrained NF-κB (p65) phosphorylation, lowered COX2 and iNOS levels, and heightened Nrf2 and HO-1 expression in BV2 cells (*P* < 0.05, Figure 4h). These outcomes uncovered that MIR17HG attenuation not only restrained microglial inflammation but also reduced the production of oxidative stressors in PD models both *in vivo* and *in vitro*.

*In vivo*: (a): Expression of Iba1 positive cells was measured by tissue immunofluorescence. (b): The profiles of IL-1β, IL-6, and TNF-α mRNA in PD mice was gauged by qRT-PCR. (c): MDA and SOD kits to quantify the content of MDA and the activity of SOD in the brain tissue of PD mice. (d). WB assayed COX2, iNOS, pNF-κB, Nrf2, and HO-1 expression in the brain tissue of PD mice. ** *P* < 0.01, *** *P* < 0.001 (vs.Sham), && *P* < 0.01, &&& *P* < 0.001 (vs.6-OHDA+Si-NC group).*In vitro*: The CM of 6-OHDA-induced SH-SY5Y cells was harvested and co-cultured with BV2 cells transfected with si-NC and si-MIR17HG for 24 hours. (e): qRT-PCR measured the MIR17HG expression in BV2. F: Il-1 β, IL-6 and TNF-α mRNA profiles were examined by qRT-PCR. G: The expression of IL-1β, IL-6, and TNF-α mRNA in the cells was tested by qRT-PCR. H: WB checked COX2, iNOS, pNF-κB, Nrf2, and HO-1 expression in BV2 cells. *P < 0.05, **P < 0.01, ***P < 0.001 (vs. Blank group), &*P* < 0.05, &&*P* < 0.01, &&&*P* < 0.001 (vs.CM^6-OHDA+Si-NC^ group).

### 3.5. MIR17HG targeted miR-153-3p

**Figure 5.**
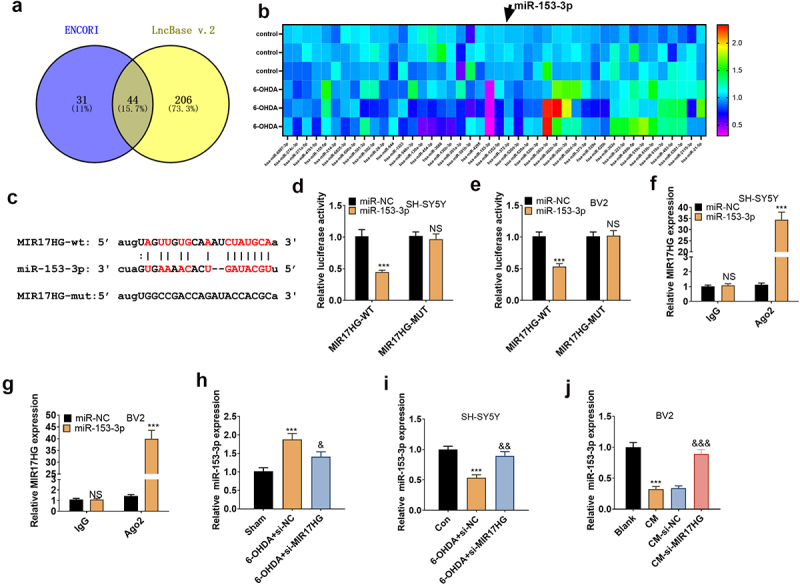
MIR17HG targeted miR-153-3p.

We scoured the ENCORI (http://starbase.sysu.edu.cn/index.php) and lncBase databases to further probe the downstream mechanism of MIR17HG and found 44 miRNAs with binding sites to MIR17HG. The expression of these 44 miRNAs in 60 OHDA-induced cells was analyzed by qRT-PCR (Figure 5a,b). In parallel, we identified that MIR17HG targeted miR-153-3p in the bioinformatics database ENCORI (Figure 5c). To clarify the targeting association between the two, we implemented a dual-luciferase reporter assay in SH-SY5Y and BV2 cells, disclosing that miR-153-3p restrained the luciferase activity of MIR17HG-WT but had no substantial influence on MIR17HG-MUT (*P* < 0.05, Figure 5d,e). A RIP test was conducted for further validation. As a result, in SH-SY5Y and BV2, the amount of MIR17HG precipitated in the Ago2 antibody group was overtly higher versus the IgG group after transfection with miR-153-3p, hinting that MIR17HG was bound to Ago2 via miR-153-3p (*P* < 0.05, Figure 5f,g). The impact of MIR17HG knockdown on miR-153-3p levels in PD mice and cellular (BV2 and SH-SY5Y) models was evaluated by qRT-PCR. The data substantiated that the 6-OHDA group had down-regulated miR-153-3p versus the Sham group *in vivo*, and the addition of si-MIR17HG lentivirus increased miR-153-3p levels (*P* < 0.05, Figure 5h). *In vitro*, miR-153-3p levels were curbed in the 6-OHDA group versus the Control group, and inhibition of MIR17HG boosted miR-153-3p profiles (*P* < 0.05, Figure 5i). Besides, the miR-153-3p profile was declined in the CM group versus the Blank group in BV2 cells, and MIR17HG attenuation heightened miR-153-3p expression (*P* < 0.05, Figure 5j). Hence, MIR17HG was a functional target of miR-153-3p, and the MIR17HG knockdown up-regulated miR-153-3p.

(a): ENCORI and lncBase databases have identified 44 miRNAs with binding sites to MIR17HG. (b): All the 44 miRNAs were analyzed by qRT-PCR in cells. (c): The predicted binding sequence between miR-153-3p and MIR17HG. (d and e): The dual-luciferase reporter assay uncovered the interaction between miR-153-3p and MIR17HG. (f and g): RIP validated the combination of miR-153-3p and MIR17HG. NS *P* > 0.05 (vs.miR-NC), *** *P* < 0.001 (vs.miR-NC). (h-j): qRT-PCR verified the expression alterations of miR-153-3p in mouse brain tissues, SH-SY5Y, and BV2 after knocking down MIR17HG. *** *P* < 0.001 (vs. Sham or Con group), & *P* < 0.05, && *P* < 0.01, &&& *P* < 0.001 (vs.6-OHDA+Si-NC or CM-Si-NC group).

### 3.6. Overexpressing miR-153-3p mitigated neuronal apoptosis and microglial inflammation

**Figure 6.**
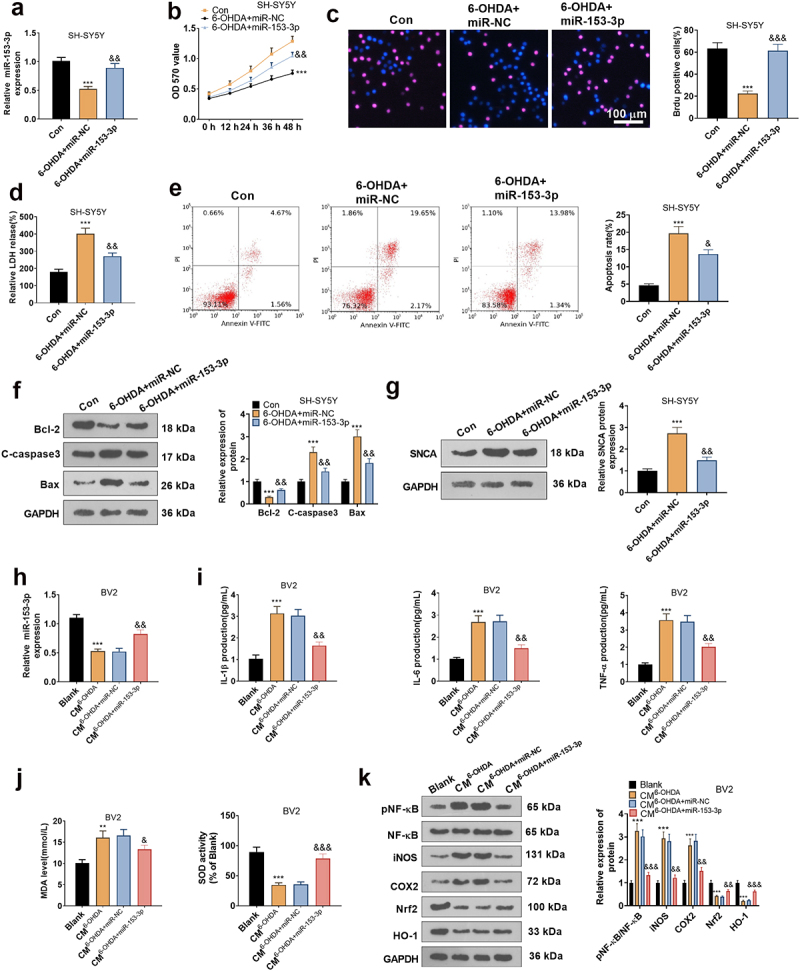
Overexpressing miR-153-3p tarnished neuronal apoptosis and microglial inflammation.

To affirm the impact of miR-153-3p on neuronal apoptosis, miR-153-3p mimics and negative controls were given in SH-SY5Y for intervention (*P* < 0.05, Figure 6a). With the utilization of MTT, BrdU staining, and LDH kits, we concluded that overexpressing miR-153-3p attenuated 6-OHDA-induced diminished cell vitality (*P* < 0.05, Figure 6b,c) and reduced LDH release (Figure 6d). FCM displayed that up-regulation of miR-153-3p depressed apoptosis versus the 6-OHDA+miR-NC group (Figure 6e). WB data testified that compared to the nonintervention group, miR-153-3p treatment brought about declined levels of C-Caspase3, Bax and SNCA and uplifted levels of Bcl2 (Figure 6f,g). Afterward, the CM of SH-SY5Y was co-cultured for 24 hours with BV2 transfected with miR-NC and miR-153-3p mimics. As demonstrated by qRT-PCR, the miR-153-3p profile was tarnished in the CM^6-OHDA^ group versus the Blank group. Transfection of miR-153-3p mimics caused elevation of miR-153-3p expression in BV2 cells versus the CM^6-OHDA+miR-NC^ group (Figure 6h). Meanwhile, qRT-PCR also affirmed that compared to the CM^6-OHDA+miR-NC^ group, the miR-153-3p intervention resulted in a depression in TNF-α, IL-1β and IL-6 expression in BV2 (*P* < 0.05, Figure 6i), along with a decrease in MDA content and facilitation in SOD activity (*P* < 0.05, Figure 6j). The NF-κB/COX2/iNOS and Nrf2/HO-1 pathways are identified to mediate PD evolvement. Here, we implemented WB and discovered that in comparison to the CM^6-OHDA+miR-NC^ group, miR-153-3p down-regulated NF-κB phosphorylation, decreased COX2 and iNOS levels, and enhanced Nrf2 and HO-1 contents (Figure 6k). Hence, overexpressing miR-153-3p suppressed neuronal apoptosis and microglial inflammation by blocking the NF-κB/iNOS/COX2 axis and activating the Nrf2/HO-1 pathway. In addition, to assay the function of the NF-κB/COX2/iNOS and Nrf2/HO-1 pathways, we applied the NF-κB inhibitor BAY 11–7082 (10 μM) and the Nrf2 inhibitor ML385 (2 μM) in 6-OHDA-induced BV2 cells, respectively. As a result, inhibition of the NF-κB axis choked 6-OHDA-induced inflammatory responses in BV2 cells versus the 6-OHDA group, but inhibition of the Nrf2 pathway stimulated the production of oxidative stress factors in BV2 cells (Supplementary Figure S1). Thus, the NF-κB/iNOS/COX2 and Nrf2/HO-1 pathways exert an essential influence on inflammation and oxidative stress.

miR-NC and miR-153-3p mimics were transfected in SH-SY5Y cells. (a): Detection of miR-153-3p expression in SH-SY5Y by qRT-PCR. (b): MTT was applied to affirm the influence of miR-153-3p overexpression on neural proliferation. (c): BrdU staining verified cell viability. (d): The LDH kit determined the level of LDH released into the cells. (e): FCM gauged cell apoptosis. (f and g): WB gauged the profiles of Bax, Bcl2, Caspase3 and SNCA. * *P* < 0.05, ** *P* < 0.01, *** *P* < 0.001 (vs.Con group), & *P* < 0.05, && *P* < 0.01, &&& *P* < 0.001 (vs.6-OHDA+miR-NC group). The CM of SH-SY5Y was co-cultured with BV2 cells transfected with miR-NC and miR-153-3p mimics for 24 hours. (h): qRT-PCR was performed to make certain the miR-153-3p expression in BV2. (i): The expression of IL-1β, IL-6 and TNF-A in BV2 cells was monitored by qRT-PCR. (j): MDA and SOD kits were employed to determine the expression of MDA and SOD in BV2 cells. (k)(: WB assayed the expression of COX2, iNOS, pNF-κB, Nrf2, and HO-1 in BV2 cells. ** *P* < 0.01, *** *P* < 0.001 (vs. Blank group), & *P* < 0.05, && *P* < 0.01, &&& *P* < 0.01 (vs.CM^6-OHDA+miR-NC^ group).

### 3.7. miR-153-3p targeted SNCA

**Figure 7.**
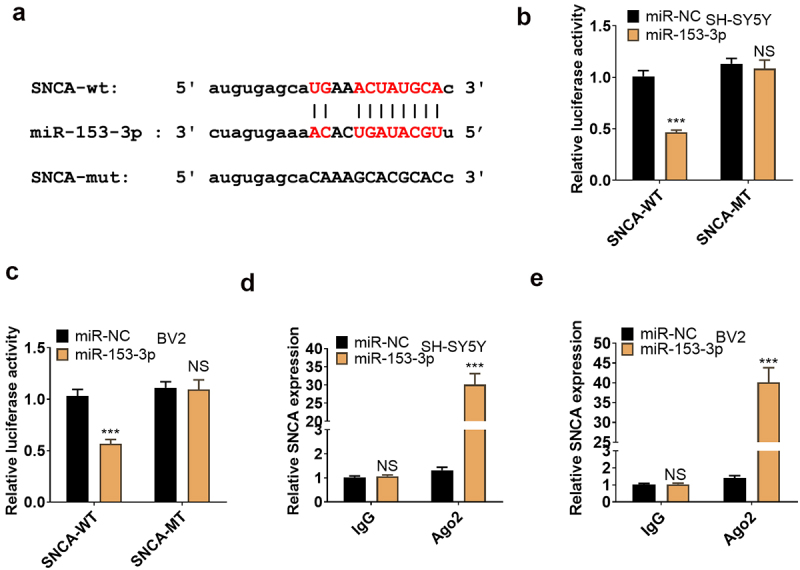
miR-153-3p targeted SNCA.

We employed the bioinformatics database ENCORI to search for downstream genes of miR-153-3p and discovered SNCA as an important one (Figure 7a). To make certain the targeting correlation between the two, we implemented a dual-luciferase reporter assay in SH-SY5Y and BV2, displaying that miR-153-3p curbed the luciferase activity of SNCA-WT but exhibited no obvious influence on SNCA-MUT (*P* < 0.05, Figure 7b,c). In parallel, a RIP test was carried out for further validation, revealing that transfection of miR-153-3p resulted in an overt higher amount of SNCA precipitated by Ago2 than IgG, hinting that SNCA was bound to Ago2 via miR-153-3p (*P* < 0.05, Figure 7d,e). Thus, SNCA was targeted by miR-153-3p.

(a): Predicted binding sequence between miR-153-3p and SNCA. (b and c): The dual-luciferase reporter assay revealed the interaction between miR-153-3p and SNCA. (d and e): RIP validated the binding association between miR-153-3p and SNCA. *** *P* < 0.001 (vs. miR-NC), NS *P* > 0.05 (vs. miR-NC).

### 3.8. Overexpressing SNCA abated the anti-inflammatory and anti-neuronal apoptotic effects of miR-153-3p

**Figure 8.**
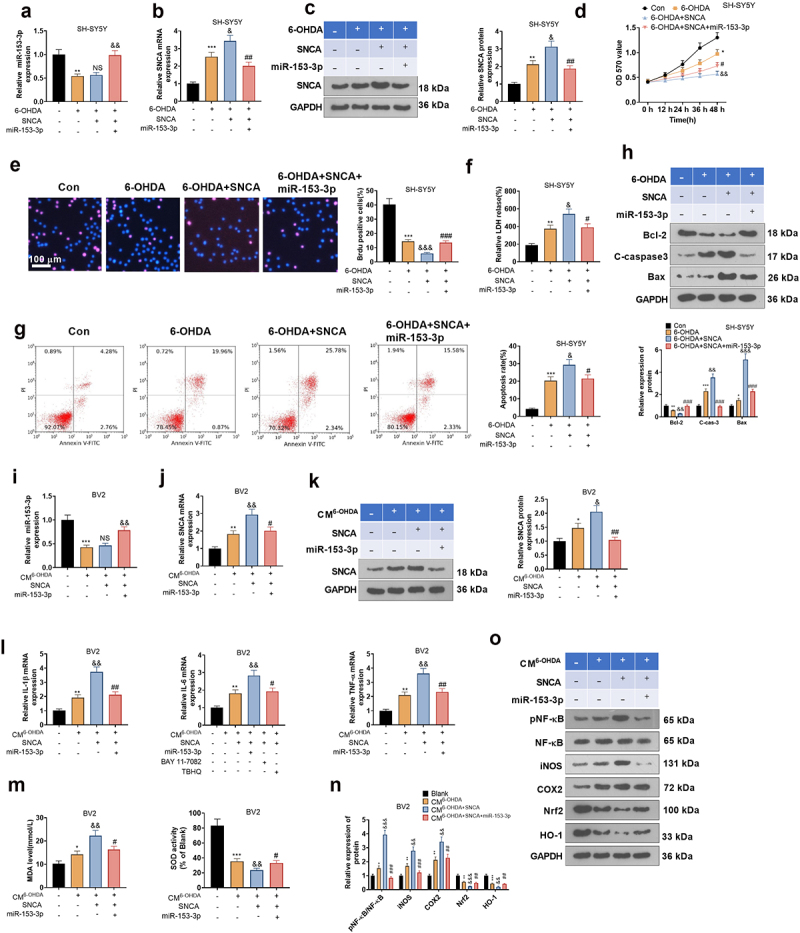
Overexpressing SNCA impaired the anti-inflammatory and anti-neuronal apoptotic effects of miR-153-3p.

The above studies suggested that miR-153-3p targeted SNCA. Nevertheless, the correlation between miR-153-3p and SNCA remains elusive. Therefore, we transfected SNCA overexpression plasmids in 6-OHDA-induced SH-SYSY5 cells and then handled the cells with miR-153-3p mimics for 24 hours. As testified by qRT-PCR and WB, overexpressing SNCA had no substantial impact on the miR-153-3p profile versus the 6-OHDA group, but it boosted the mRNA and protein expression of SNCA. In parallel, up-regulating miR-153-3p led to a facilitated expression of miR-153-3p and weakened expression of SNCA in cells versus the 6-OHDA+SNCA group (*P* < 0.05, Figure 8a-c). Functional experiments manifested that overexpressing SNCA curbed neuronal cell viability (*P* < 0.05, Figure 8d,e) and triggered LDH release from neurons and intensified neuronal apoptosis (*P* < 0.05, Figure 8f-h) versus the 6-OHDA group. Meanwhile, up-regulation of miR-153-3p attenuated SNCA-induced cell viability inhibition and neuronal apoptosis versus the 6-OHDA+SNCA group.

We transfected 6-OHDA-induced SH-SYSY5 cells with SNCA overexpression plasmids and then handled them with miR-153-3p mimics for 24 hours. (a and b): qRT-PCR tested miR-153-3p and SNCA expression in SH-SY5Y cells. (c): SNCA expression in SH-SY5Y cells was gauged by WB. (d): MTT verified SH-SY5Y cell viability. (e): BrdU assay was employed for counting the BrdU-positive cell number in SH-SY5Y cells. (f): The LDH kit was applied to examine the release of LDH from SH-SYSY cells. (g): Cell apoptosis was estimated by FCM. (h): WB was conducted for evaluating the expression of Bax, C-caspase-3 and Bcl-2 in SH-SY5Y cells. * *P* < 0.05, ** *P* < 0.01, *** *P* < 0.001 (vs. Con group), & *P* < 0.05, && *P* < 0.01, &&& *P* < 0.01 (vs.6-OHDA group). NS *P > *0.05, # *P* < 0.05, ## *P* < 0.01, ### *P* < 0.01 (vs.6-OHDA+SNCA group).In addition, we harvested CM from SH-SY5Y cells treated as described above and incubated the CM with BV2 cells for 24 hours. (i and j): qRT-PCR gauged miR-153-3p expression in BV2 cells. (k): SNCA profiles in BV2 cells were assayed by WB. (l): The levels of IL-1β, IL-6, and TNF-α in BV2 cells were compared by qRT-PCR. (m): The oxidative stress kit was employed to assess MDA expression and SOD activity in BV2 cells. (n and o): WB detection of protein expression of COX2, iNOS, pNF-κB, Nrf2, and HO-1 in BV2 cells. * *P* < 0.05, ** *P* < 0.01, *** *P* < 0.001 (vs. Blank group), & *P* < 0.05, && *P* < 0.01, &&& *P* < 0.01 (vs.CM^6-OHDA^ group). # *P* < 0.05, ## *P* < 0.01, ### *P* < 0.01 (vs.CM^6-OHDA+SNCA^ group).

Additionally, we gathered CM from SH-SY5Y cells treated as described above and cultured the CM with BV2 cells for 24 hours. The expression of miR-153-3p and SNCA in BV2 cells was measured using qRT-PCR and WB, and the outcomes were consistent with those in SH-SYSY cells (*P* < 0.05, Figure 8i-k). Also, we gauged the expression of inflammatory factors and oxidative stressors in BV2 cells with qRT-PCR and an oxidative stress kit. Notably, by contrast with the CM^6-OHDA^ group, CM^6-OHDA+SNCA^ heightened the expression of IL-1β, IL-6, TNF-α and MDA and hindered the SOD activity in cells. Up-regulating miR-153-3p suppressed the expression of inflammatory factors and MDA and enhanced SOD activity in BV2 cells versus the CM^6-OHDA+SNCA^ group (*P* < 0.05, Figure 8l,m). As testified by WB outcomes, compared to the CM^con^ group, CM^6-OHDA+SNCA^ choked the expression of HO-1 and Nrf2, elevated the protein expression of iNOS and COX2 and heightened the phosphorylation of NF-κB in the cells. Compared to the CM^6-OHDA+SNCA^ group, up-regulating miR-153-3p brought about elevated protein expression of HO-1 and Nrf2, declined expression of iNOS and COX2, and weakened phosphorylation of NF-κB in BV2 cells (*P* < 0.05, Figure 8n,o).

### 3.9. MIR17HG attenuated anti-inflammatory and anti-neuronal apoptotic effects of miR-153-3p

**Figure 9.**
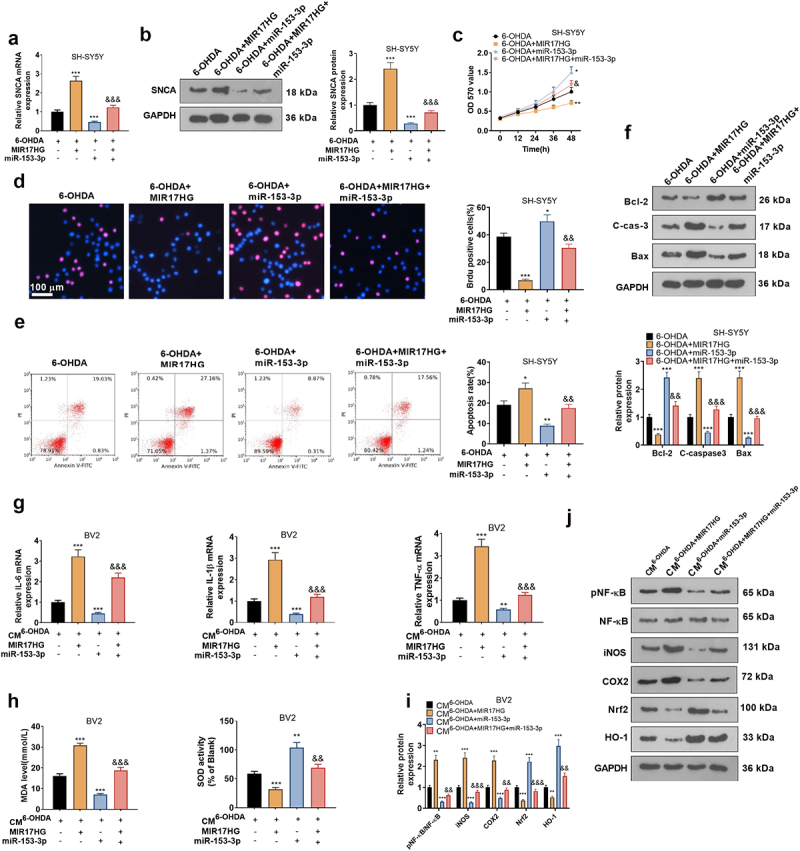
MIR17HG hindered the anti-inflammatory and anti-neuronal apoptotic properties of miR-153-3p.

To determine whether MIR17HG acts through regulation of miR-153-3p, we transfected MIR17HG overexpression plasmids and miR-153-3p mimics alone or in combination into 6-OHDA-treated SH-SY5Y cells. qRT-PCR and WB outcomes illustrated that up-regulation of MIR17HG elevated SNCA levels versus the 6-OHDA group, while miR-153-3p exerted the reverse effect. The addition of MIR17HG to the 6-OHDA+miR-153-3p group raised the SNCA expression (*P* < 0.05, Figure 9a,b). With the application of MTT and BrdU staining, we discovered that cell viability in the 6-OHDA+MIR17HG+miR-153-3p group was choked versus the 6-OHDA+miR-153-3p group (Figure 9c,d). Besides, FCM and WB results disclosed that the addition of MIR17HG to the 6-OHDA+miR-153-3p group resulted in facilitated levels of Bax and C-caspase-3 and declined Bcl-2 profiles in SH-SY5Y cells (Figure 9e,f). Hence, MIR17HG dampened the anti-apoptotic effect of miR-153-3p on neurons. Next, we transfected MIR17HG overexpression plasmids and miR-153-3p mimics individually or together into BV2 cells and co-cultured CM of 6-OHDA-induced SH-SY5Y cells with BV2 cells for 24 hours. As displayed by qRT-PCR, the expression of IL-1β, IL-6, TNF-α was elevated (*P* < 0.05, Figure 9g), MDA was up-regulated and SOD activity was restrained (*P* < 0.05, Figure 9h) in BV2 in the CM^6-OHDA+MIR17HG-miR−153−3p^ group versus the CM^6-OHDA+miR−153−3p^ group. Finally, the profiles of the NF-κB/iNOS/COX2 and Nrf2/HO-1 pathways were monitored by WB, which displayed that the CM^6-OHDA+MIR17HG-miR−153−3p^ group had curbed levels of Nrf2 and HO-1, amplified contents of COX2 and iNOS, and heightened phosphorylation of NF-κB versus the CM^6-OHDA+miR−153−3p^ group (*P* < 0.05, Figure 9i,j). Therefore, MIR17HG curbed anti-inflammatory and anti-neuronal apoptotic effects of miR-153-3p.

MIR17HG overexpression plasmids, miR-153-3p mimics, and MIR17HG overexpression plasmids+miR-153-3p mimics were transfected into 6-OHDA-treated SH-SY5Y cells, respectively. (a and b): qRT-PCR tested the SNCA expression in SH-SY5Y cells. (c): MTT verified SH-SY5Y cell viability. (d): BrdU assay was employed for counting the number of positive cells for BrdU in SH-SY5Y cells. (e): Cell apoptosis was monitored by FCM. (f): WB was conducted for evaluating the expression of Bax, C-caspase-3 and Bcl-2 in SH-SY5Y cells. * *P* < 0.05, ** *P* < 0.01, *** *P* < 0.001 (vs.6-OHDA group), & *P* < 0.05, && *P* < 0.01, &&& *P* < 0.01 (vs.6-OHDA+miR-153-3p group). The CM of SH-SY5Y was co-cultured with BV2 cells transfected with MIR17HG overexpression plasmids, miR-153-3p mimics, and MIR17HG overexpression plasmids + miR-153-3p mimics for 24 hours. (g): The profiles of IL-1β, IL-6, and TNF-a were assessed in BV2 cells by qRT-PCR. (h): MDA and SOD kits were adopted to analyze the expression of MDA and SOD in BV2 cells. (i and j): WB assayed COX2, iNOS, pNF-κB, Nrf2, and HO-1 expression in BV2 cells. ** *P* < 0.01, *** *P* < 0.001 (vs.CM^6-OHDA^), & *P* < 0.05, && *P* < 0.01, &&& *P* < 0.001 (vs.CM^6-OHDA+miR−153−3p^ group).

## 4. Discussion

PD is a chronic neurodegenerative disorder that features resting tremor, rigidity, bradykinesia and postural instability [39]. Microglial activation exerts its neurotoxic effects through the generation of pro-inflammatory cytokines and an increase in oxidative stress, which is the main mechanism of PD [40]. MDA and SOD are markers of oxidative stress and antioxidants. When redox homeostasis is lost in the brain, oxidative stress can lead to severe damage, resulting in neuronal loss [41]. IL-1β, IL-6, and TNF-α mediate neuroinflammation [42]. In this study, 6-OHDA-induced mice and SH-SY5Y cells were applied for *in-vivo* and *ex-vivo* PD models. Our results corroborated that 6-OHDA hindered SH-SY5Y cell viability. In addition, 6-OHDA treatment boosted LDH release, increased Caspase3 levels and the production of oxidative stress as well as the inflammatory response of microglia. In summary, our data hint that 6-OHDA treatment contributes to SH-SY5Y cell injury.

Over the past few years, the involvement of lncRNAs in the pathology of PD has attracted increasing interest [43,44]. For example, in MPP + treated SH-SY5Y cells, the long noncoding RNA SRY-box transcription factor 2 overlapping transcript was up-regulated, and its inhibition suppressed the contents of TNF-α, IL-1β and ROS and heightened SOD activity [8]. Additionally, LncRNA NEAT1 is highly expressed in MPP+-treated SH-SY5Y cells, and inhibition of LncRNA NEAT1 boosts cell viability, suppresses cytotoxicity and weakens inflammatory responses by modulating miR-124-3p and PDE4B expression levels [45]. lncRNA MIR17HG is widely expressed in cancer, which has implications with cervical cancer [46] and osteosarcoma [47] as an oncogene. Nevertheless, the potential mechanisms by which lncRNA MIR17HG contributes to PD remain unclear. Here, using a model of SH-SY5Y cells intervened by 6-OHDA, we observed that the MIR17HG level was uplifted in SH-SY5Y cells and that inhibition of MIR17HG had a protective effect on cell viability. 6-OHDA treatment intensified SH-SY5Y cell apoptosis, the production of oxidative stress and the release of inflammatory factors, and these pathological changes can be attenuated by MIR17HG inhibition.

6-OHDA is among the most familiar drugs to build PD animal models in recent years. The most commonly used method is the unilateral injection of 6-OHDA into the SN or MFB, which causes rapid death of DA neuronal cells in a shorter time course, creating an acute PD model similar to that triggered by MPTP. Microglial deficiency causes PD-like symptoms in mice, featured by impaired motor coordination and cognitive learning, loss of neurons in TH, increased neuroinflammation and reduced dopamine levels in the striatum [48]. Here, we set up a 6-OHDA-induced PD mouse model and discovered that 6-OHDA triggered behavioral changes, a massive reduction in TH + cells and an increase in neuronal apoptosis in PD mice, along with an up-regulation of SNCA.

miRNAs are noncoding RNAs whose altered levels have been demonstrated in different PD models and in the human brain. For example, down-regulation of miR-217 or miR-138-5p curbs MPP-induced inflammation and oxidative stress as well as neuronal apoptosis in SH-SY5Y cells, thereby contributing to PD [49]. miR-29c protects against neuroinflammation and apoptosis in PD via directly targeting SP1, thus subserving PD diagnosis and treatment [50]. miR-153-3p, a miRNA, exerts a neuroprotective role in diversified human nervous system diseases. For instance, in PD, LncRNA SNHG1 restrains the viability and strengthens the apoptosis of MPP-treated SH-SY5Y cells by stimulating miR-153-3p to regulate the PTEN/AKT/mTOR axis [15]. Several cell signaling pathways, including the NF-κB/iNOS/COX2 and Nrf2/HO-1 pathways, are also involved in inflammatory and oxidative stress responses in the nervous system. For example, Centipeda minima exhibit anti-neuroinflammatory properties *in vivo* and *in vitro* by inactivating the NF-κB/iNOS/COX2 axis and dampening the generation of pro-inflammatory mediators [51]. In parallel, Danshensu resists oxidative stress by motivating the PI3K/AKT/Nrf2 pathway and facilitating Nrf2-induced expression of HO-1 in PD [52]. Our current outcomes disclosed that the miR-153-3p level was choked in 6-OHDA-induced SH-SY5Y cells. LPS treatment activated microglia, leading to an increase in the inflammatory marker iNOS and directly resulting in the activation of COX-2. Co-culture of BV2 with SH-SY5Y cells revealed that miR-153-3p overexpression mitigated 6-OHDA-induced neuronal apoptosis and microglial inflammation and oxidative stress, blocked the NF-κB/iNOS/COX2 pathway and spurred the Nrf2/ HO-1 axis in SH-SY5Y cells. All these outcomes imply a protective effect of miR-153-3p against 6-OHDA-induced SH-SY5Y cell damage.

α-syn, a small protein, is a product gene of SNCA. The accumulation of α-syn is a pathological hallmark of PD and is associated with the reduced neuronal length and axonal degeneration in midbrain dopaminergic neurons [16]. Knockdown of α-syn alleviates MPP(+)-induced mitochondrial dysfunction in SH-SY5Y cells [53]. Meanwhile, amide-bridged nucleic acid (AmNA)-modified antisense oligonucleotides (ASO) targeting α-syn ameliorates the neurological deficits of PD and can be an underlying therapeutic option for SNCA-related pathology in PD [54]. Emerging reports testified that lncRNAs can act as ceRNAs to sponge miRNAs, thereby regulating α-syn and influencing disease progression. For example, LncRNA SNHG1 activates SIAH1 in SH-SY5Y cells by targeting miR-15b-5p, thereby enlarging the aggregation and toxicity of α-syn [55]. Those studies uncovered that non-coding RNAs affect SNCA expression and get involved in PD progression. Here, we performed a dual-luciferase reporter assay and Pearson correlation analysis, which exhibited that the expression of miR-153-3p was reversely linked to that of MIR17HG and SNCA. We up-regulated miR-153-3p on the basis of overexpression of SNCA in SH-SY5Y cells treated with 6-OHDA. It was also found that up regulation of miR-153-3p inhibited the expression of SNCA in cells and inhibited the proliferation inhibitive and pro-apoptotic effects of SNCA (Figure 8), suggesting that miR-153-3p influences neuronal apoptosis and microglial inflammation through targeting SNCA. Overall, these outcomes testified that MIR17HG modulated miR-153-3p in a targeted manner and thus affected the expression of SNCA, thereby influencing PD evolvement.

Several limitations should be addressed in our future studies. First, the regulatory effects of MIR17HG-miR-153-3p-SNCA axis should be confirmed in PD model *in vivo*. Second, larger clinical samples are needed for evaluating the predictive role of the MIR17HG-miR-153-3p-SNCA axis in PD development.

## 5. Conclusion

Our research confirms that MIR17HG displayed pronounced expression in PD tissues and cells. Attenuation of MIR17HG expression reduces neuronal apoptosis and inflammatory responses and oxidative stress in microglia in PD models both *in vivo* and *in vitro* and regulates PD evolvement by modulating the miR-153-3p/SNCA axis. Our research may contribute to the development of early diagnosis and treatment options for PD.

## Supplementary Material

Supplemental Material

## Data Availability

The data sets used and analyzed during the current study are available from the corresponding author on reasonable request.
