# Supplementary figures and images for: LncRNA miR-17-92a-1 cluster host gene (MIR17HG) promotes neuronal damage and microglial activation by targeting the microRNA-153-3p/alpha-synuclein axis in Parkinson’s disease
Source: Bioengineered. 2022 Feb 9;13(2):4493–516. doi: 10.1080/21655979.2022.2033409 (PMC8974023; doi:10.1080/21655979.2022.2033409)

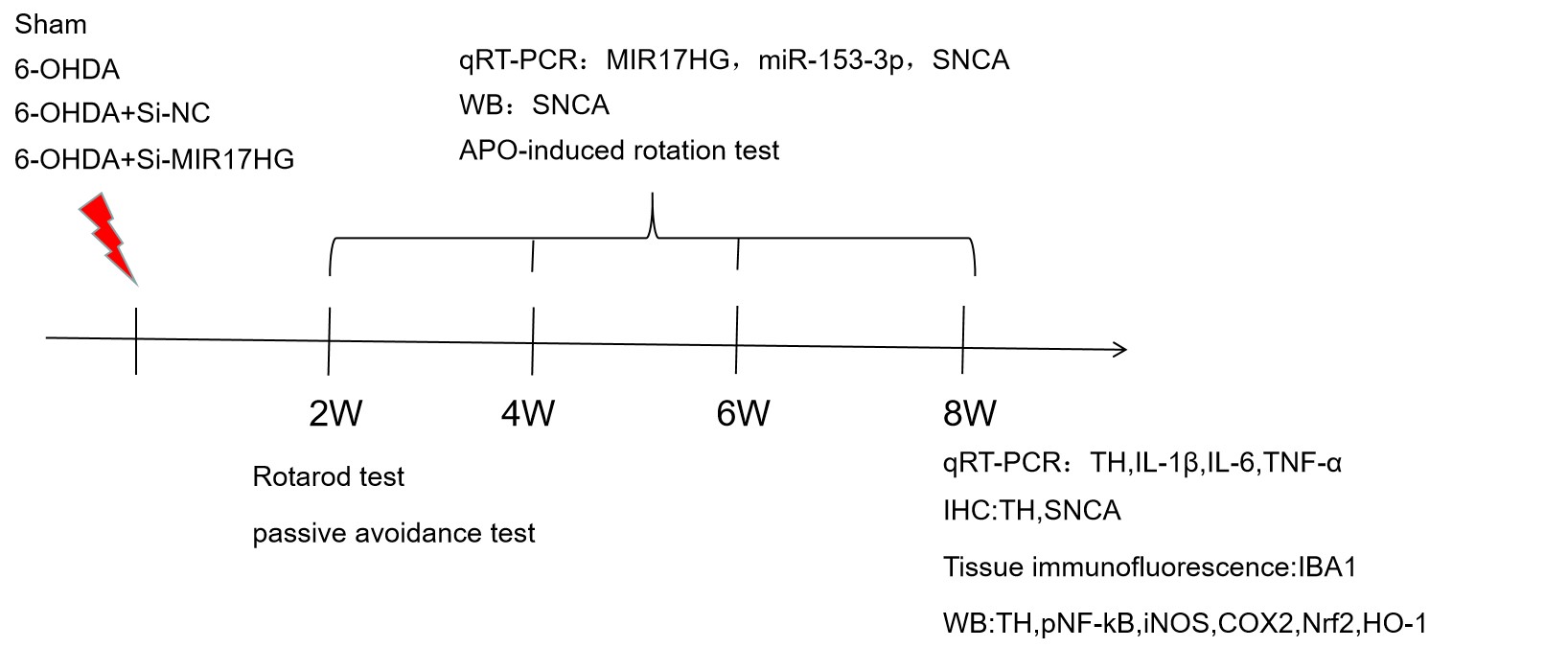

Supplement: Supplemental Material [file KBIE_A_2033409_SM8420.zip › supplementary/Sup figure1.jpg]

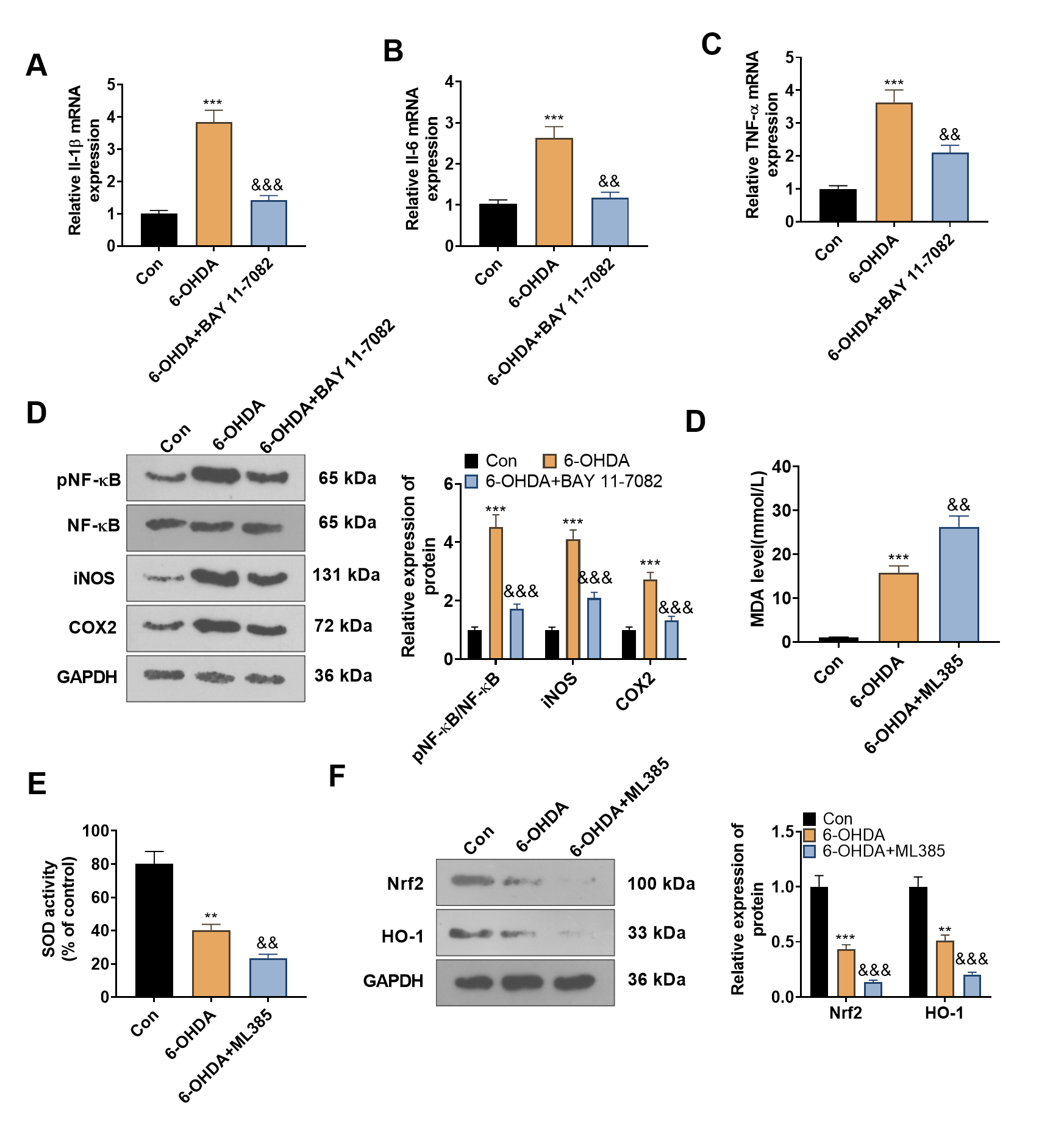

Supplement: Supplemental Material [file KBIE_A_2033409_SM8420.zip › supplementary/Sup figure2.tif]
